# Endoscopic nipple sparing mastectomy with immediate implant-based reconstruction versus breast conserving surgery: a long-term study

**DOI:** 10.1038/srep45636

**Published:** 2017-03-31

**Authors:** Junze Du, Quankun Liang, Xiaowei Qi, Jia Ming, Jing Liu, Ling Zhong, Linjun Fan, Jun Jiang

**Affiliations:** 1 Breast surgery, Southwest Hospital, the Third Military Medical University, Chongqing 400038, China; 2 Head-neck surgery, Tumor Hospital, Guangxi Medical University, Guangxi, 530021, China; 3 Three glands surgery, the Second Affiliated Hospital, Chongqing Medical University, Chongqing 400010, China

## Abstract

To evaluate the differences between endoscopic nipple sparing mastectomy (ENSM) with immediate implant-based reconstruction and breast conserving surgery(BCS) applied to early-stage breast cancer in postoperative outcomes, function, and cosmesis. we made a prospective, non-randomized study reviewed a total of 346 cases of breast cancer from January 2007 to December 2011, including 189 cases of BCS and 157 cases of ENSM. All the patients were followed up to April 2016, with a median follow-up time of 74 months. The operative time, blood loss and drainage, postoperative complications, postoperative cosmesis, local recurrence rate, disease-free survival rate and overall survival rate of the two groups were compared. we found out that the operative time of ENSM was longer than that of BCS. There was no difference in blood loss and drainage, the postoperative complications, the disease-free survival rate and overall survival rate between the two groups. In regarding to cosmesis, patients in the ENSM group were more likely to get a satisfactory postoperative breast appearance. we reached a conclusion that ENSM is a safe and effective operative method retainingadvantages of TSSM to further improve the postoperative cosmetic effect, without increasing other risks. The surgery provides a new choice for patients with early-stage breast cancer.

The morbidity associated with breast cancer, which is the most common malignant disease in females, is rising annually at an alarming rate^1^,^2^. Breast cancer is associated with serious physical and mental ailments. In recent years, the overall survival rate of breast cancer has dramatically improved because of therapeutic advances and comprehensive care^3^. Breast conservative surgery (BCS) is the standard of care for early breast cancer (EBC). The long-term survival rates do not vary between early breast cancer patients receiving BCS and traditional modified radical mastectomy^4^. In the USA, BCS accounts for more than 60%^5^ of all surgeries, while it is less than 10% in China. Even in big cities of China, the rate is only 24%^6^,^7^. The possible factors underlying the limited usage of BCS include concerns involving local recurrence and fear of radiotherapy after breast surgery^8^.

The advent of total skin-sparing mastectomy (TSSM) and breast reconstruction offers an alternative for patients contraindicated for BCS. Mammectomy, which spares skin, nipple and areola, entails similar invasive surgery as traditional BCS, and insures safety and effectiveness through long-term follow-up^9^,^10^,^11^,^12^. Moreover, it maximally improves breast aesthetics and quality of life via a single-stage reconstruction. To further enhance the cosmetic effect, we investigated a new method of operation known as endoscopic nipple sparing mastectomy (ENSM) with immediate implant-based reconstruction. We compared it with BCS to analyze the differences in therapeutic outcomes.

## Patients and Methods

The study was approved by the ethics committee of Southwest Hospital of Third Military Medical University (permission number: SWH2006B012). This study was carried out in accordance with the approved guidelines at Southwest Hospital. Informed consent was obtained from all the patients. In this study, we reviewed a total of 346 cases of breast cancer from January 2007 through December 2011 including 189 cases undergoing BCS and 157 cases treated with ENSM via immediate implant-based reconstruction. All patients were fully aware of the differences between the two types of surgery to select the procedure most appropriate for their condition.

The inclusion criteria were as follows: 1) females, ages 18 to 60 years; 2) unilateral invasive breast cancer confirmed by preoperative core needle biopsy; 3) absence of deviation or retraction of the surface of the skin, nipple and areola of breast and chest; 4) tumor ≤3 cm in size or ≤3 cm in diameter after neoadjuvant chemotherapy; and 5) no obvious enlargement of axillary lymph nodes and fusion. ENSM was performed under the following conditions: 1) breast skin without peel syndrome or dimple sign; 2) small breast volume measured using the drainage method before surgery (less than 400 mL); and 3) breast without ptosis. Nonetheless, patients undergoing BCS met the following criteria: 1) tumor location more than 2 cm away from the nipple areola margin; 2) absence of multicentric lesions suggested by preoperative ultrasound or mammography; 3) absence of history of breast radiotherapy; and 4) signs of improved appearance according to tumor size and breast volume.

### Preoperative adjuvant therapy

Patients receiving neoadjuvant therapy were eligible for the study. Neoadjuvant chemotherapy included a TE regimen: docetaxel 75 mg/m^2^, epirubicin 75 mg/m^2^; cycled every 14 days for 2–4 cycles.

### Surgical procedures

BCS: If the nidus was located in the upper side of the breast, an arc incision centering on the nipple was made. However, if it was located in the inner or outer range, a Langer’s incision was performed. If the nidus was in the lower range, a radial incision was created. A small portion of normal surrounding breast tissue was removed with the tumor. If the tumor resection margin was present at a distant edge greater than10 mm, the excision range included needle biopsy. If positive margin was present after obtaining the frozen tissue, it was excised again according to the criteria for sentinel biopsy^13^,^14^. Axillary sentinel lymph node biopsy was performed initially. Axillary lymph node dissection was performed if the frozen section examination showed positive results.

#### ENSM

**Figure 1:**
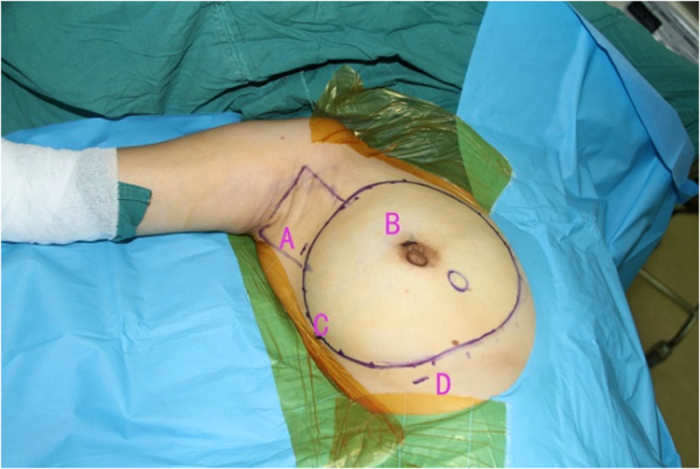
Pre-operation mark the edge of breast, location of breast, axillary range and location of incision.

**Figure 2:**
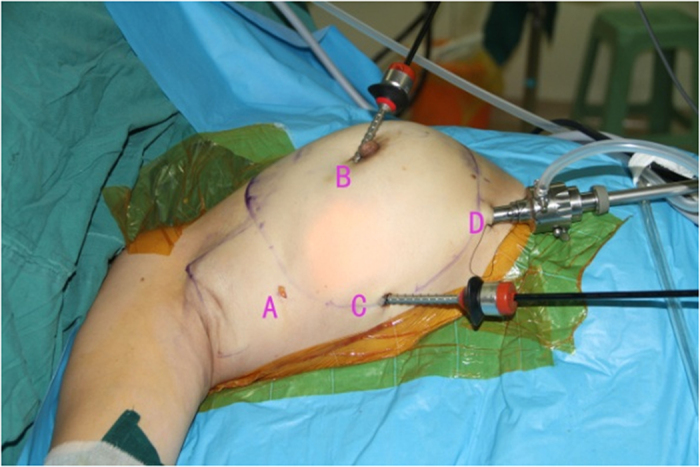
When perform the ALND, D is used for view port, B C port is used for operate.

**Figure 3:**
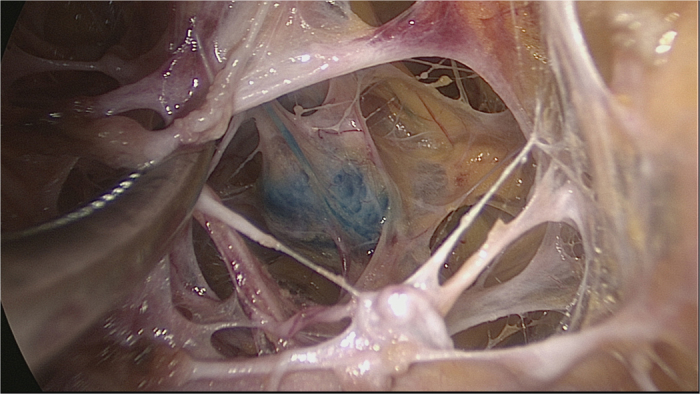
Axillary lymph node of superficial layer of can be identifited directly after liposuction.

**Figure 4:**
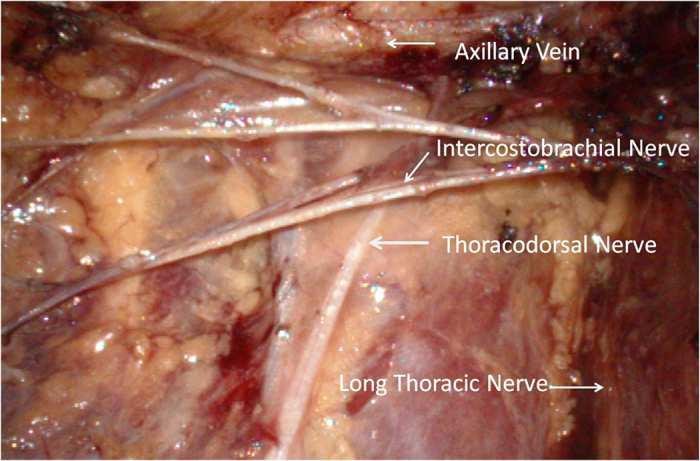
Axillary vein, long thoracic nerve, thoracodorsal nerve, intercostobrachial nervecan be identifited clearly after ALND.

**Figure 5:**
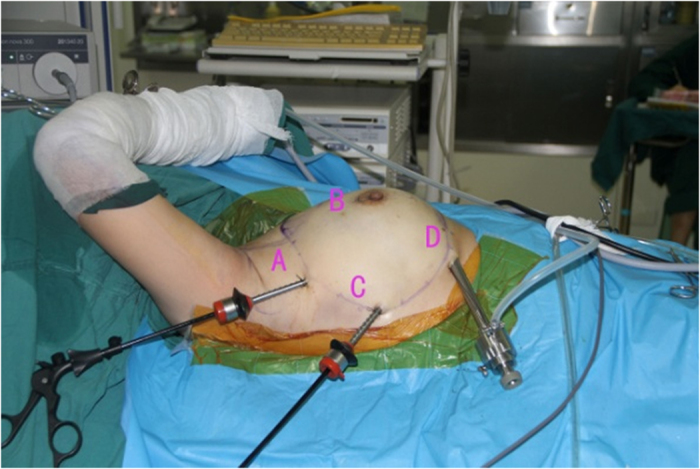
When perform the ENSM, A and C is used for operate port, D is used for view port.

**Figure 6:**
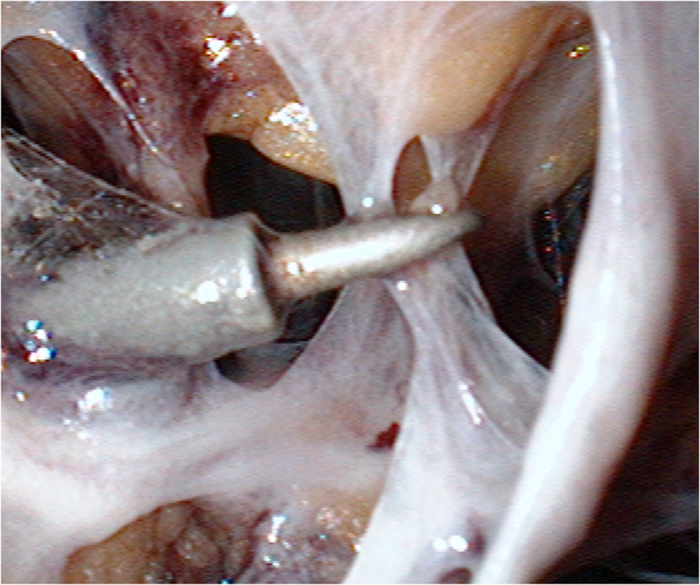
After liposuction, There were only Cooper ligaments left branching out through and around breast tissue to the dermis of the skin overlying the breast skin in the subcutaneous space, cut off the cooper ligament and enlarge the space.

1. Endoscopic approach: The edge of breast and the range of axilla were marked, and a 1-cm incision was made at the outer upper edge of breast, near the axilla (A); outer upper edge of areola (B); 1 cm from the outer edge of breast at the nipple level (C); 1 cm from the outer lower edge of breast along the anterior axillary line (D), respectively (Fig. 1). Incisions A, C and D were used for removal of mammary glands, and incisions B, C and D were used for dissection of axillary lymph nodes.
2. Lipolysis and liposuction: A fat-soluble liquid was prepared using 250 mL of sterilized distilled water, 250 mL of normal saline, 20 mL of 2% lidocaine, and 1 mL of 0.1% epinephrine. The mixture was injected subcutaneously into breast, retromammary space and axilla through incisions A, B, C and D. After 10 min, liposuction was performed subcutaneously via incisions created in the breast, retromammary space and axillary area.
3. Endoscopic sentinel lymph node biopsy: Patients eligible for sentinel lymph node biopsy were injected with ^99m^Tc-ASC (antimony trisulfide colloid) in the areola area 24 h before operation, and with methylene blue (10 mL) in the same area 10 min before operation, followed by endoscopic sentinel lymph node excision. If the intra-operative frozen section analysis showed positive results, endoscopic axillary dissection was performed subsequently.
4. Endoscopic axillary dissection (Figs 2, 3, 4): Trocars filled with CO_2_ (pressure:10 mm Hg) were inserted through incisions B, C and D, and the Cooper’s ligament was cut off in front of the trocars. The axillary dissection was performed in the operation zone of the axilla.
5. Endoscopic mammary gland excision (Figs 5 and 6): Mammary gland excision was performed in the following sequence: outer lower quadrant - outer upper quadrant - inner lower quadrant - inner upper quadrant.
6. Mammary gland removal and prosthesis implantation: Incision A was extended to 5 cm, to remove the mammary gland and axillary tissue, and the operation zone was flushed with distilled water. The mammary gland was weighed and suitable prosthesis (160 g~315 g) was selected for implantation into the posterior gap of ectopectoralis. Before implantation, the subpectoral space was separated. The lower attachment point of pectoralis major was sectioned off to ensure that the lower edge of the prosthesis reached the submammary fold. The muscle fascia in front of the pectoralis major were intact during the gland resection, to ensure that the major part of the prosthesis was posterior to pectoralis major, and the lower part was located under the muscle fascia. After implantation, a drainage tube was placed, and the incision was finally sutured.

### Postoperative adjuvant therapy

Drainage was performed from day 5 to day 7 after surgery. The drainage tubes were removed when the drainage was less than 10 mL. Postoperative chemotherapy was administered to patients for 1 to 2 weeks, similar to preoperative chemotherapy. Treatment was administered when the white blood cell count was lower than normal level. Patients in the BCS group underwent breast radiation therapy following chemotherapy unlike those in the ENSM group. Patients also required parasternal, axillary and supraclavicular radiotherapy when postoperative pathological results indicated metastasis of more than 3 axillary lymph nodes.

Targeted therapy was administered to patients with postoperative pathological and immunohistochemical staining indicating Her-2+++ or Her-2++ and testing positive with fluorescence *in situ* hybridization (FISH). The initial dose of trastuzumab was 8 mg/Kg, followed by 6 mg/kg, cycled every 21 days for 1 year.

After chemotherapy (or radiotherapy), all the patients were followed up in the outpatient department every 3 to 6 months. Patients who failed to attend the out-patient department for follow-up were contacted using questionnaires by mail or over the telephone.

### Surgical outcomes

Surgical outcomes included operative time, intraoperative blood loss, postoperative drainage and postoperative complications (infection, marginal necrosis of incision and upper extremity edema). Nipple necrosis and prosthesis fixation during endoscopic surgery were closely monitored. Furthermore, cosmesis was assessed by the surgeon and patients together as follows:

1. Excellent: The reconstructed and the opposite breasts were perfectly symmetrical and equal in size. The patients were extremely satisfied.
2. Good: The reconstructed breast was equal in size but a little higher than the opposite one. However, there was no significant difference after dressing. The patients were satisfied.
3. Mediocre: Bilateral breasts were asymmetric distinctly without any significant difference after dressing. The patients were dissatisfied.
4. Poor: The reconstructed breast was out of shape. The patients were extremely dissatisfied.

Meanwhile, the rates of local recurrence and distant metastases, and mortality were also considered when assessing the surgical outcomes.

### Statistical analysis

The groups were compared using the X2 test, Mann–Whitney U test, Student’s t test, or Fisher’s exact test for qualitative or quantitative variables, and when appropriate, the SPSS software version 19.0 (SPSS Inc, Chicago, IL,USA) was used. A p value less than 0.05 was considered statistically significant.

## Results

No significant differences in age, hormone receptor status, pathological types, blood loss, postoperative drainage, lymphatic metastasis and TNM stage were found between the BCS and ENSM groups. The operative time in the ENSM group was longer than in the BCS group (p < 0.05). A total of 140 patients in the BCS group and 52 in the ENSM group underwent sentinel lymph node biopsy (p < 0.05). In the BCS group, all the patients were treated with breast radiation therapy, and 14 patients (7.41%) received axillary, parasternal and supraclavicular radiation therapy. Most patients in the ENSM group were not exposed to radiation therapy except 18 (11.46%) who received axillary, parasternal and supraclavicular radiation (Table 1).

We found no significant differences in infection rate (4.23% vs. 3.18%), upper limb lymphedema (12.17% vs. 9.55%) and marginal necrosis of incison (8.47% vs. 7%) between BCS and ENSM groups (p > 0.05) (Table 2). In the ENSM group, partial nipple necrosis occurred after operation in 7 patients (4.46%). Capsular contraction of prosthesis occurred in 21 patients during 10–14 months postoperatively, and 2 of 21 were ranked grade III, according to Baker’s grading system, while the other patients were grade I/II.

**Figure 7:**
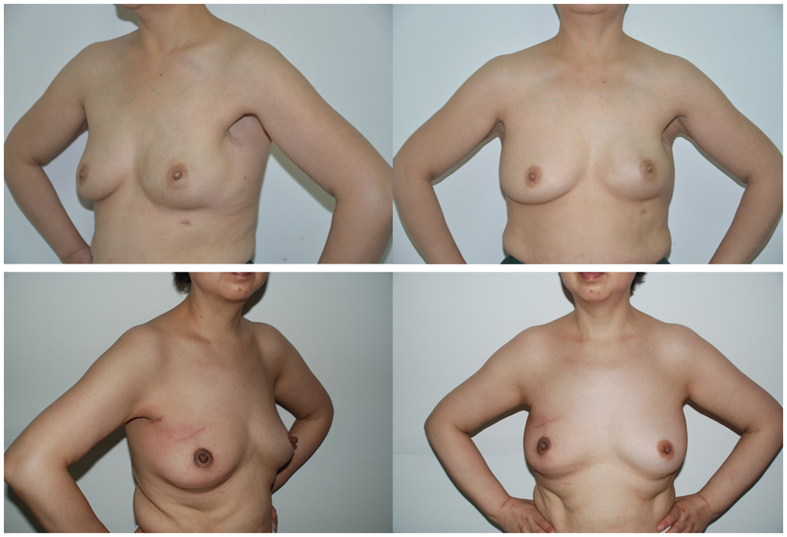
Cosmesis outcome of ENSM(up): a 49 years old female patients with early stage breast cancer. 1.5 years after receiving ENSM plus immediate implant-based reconstruction, anterior view (left), Lateral view(right) versus BCS(down): a 38 years old female patients with early stage breast cancer. 1 year after receiving breast conserving surgery, anterior view (left).

Comparing cosmetic effects between the BCS and ENSM group (Table 3), we found a better outcome in the ENSM group compared with the BCS group (69.43% vs. 58.2%) as endoscopic surgery was performed via small incisions. Most of the incisions in the breast region were inconspicuous (Fig. 7), with no significant difference between the two groups (p > 0.05).

**Figure 8:**
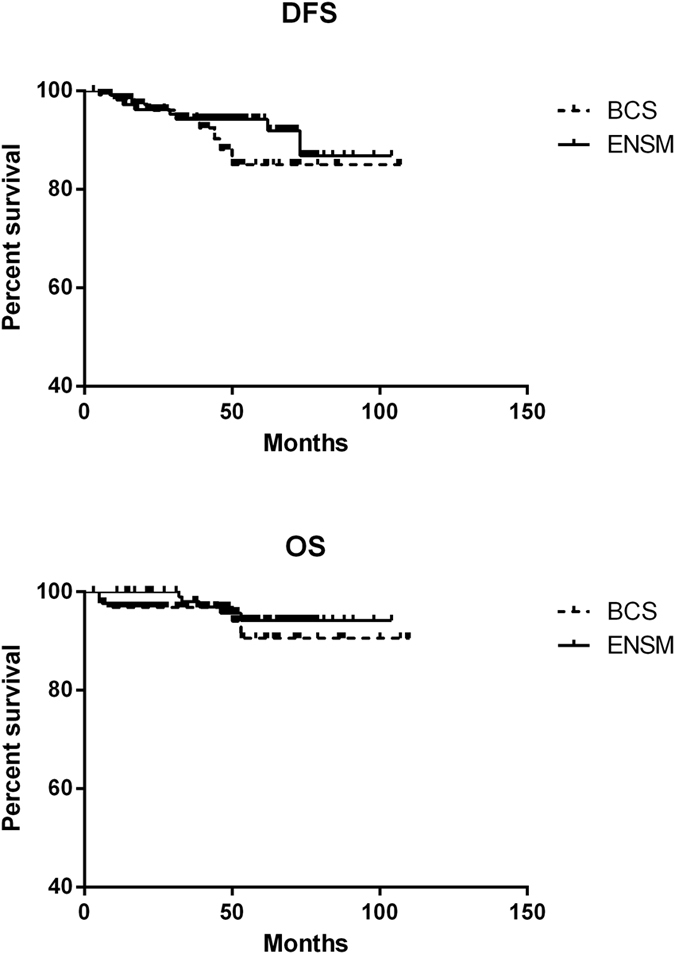
Comparison of Overall Survival (OS) and Disease Free Survival (DFS) between two groups.

In the follow-up range from 52 months to 111 months (median follow-up time: 74 months) of all patients, distant recurrence in metastasis occurred in 7 cases of ENSM group, without local recurrence, but 5 deaths. Recurrent metastasis occurred in 9 cases of BCS group, including 6 cases of local recurrence, and 6 deaths. The disease-free survival (DFS) and overall survival (OS) in the BCS group were all lower than in the ENSM group, without any significant difference (p > 0.05) (Fig. 8).

## Discussion

BCS is a modified procedure indicated for early-stage breast cancer, with results similar to those of radical mastectomy^4^. However, in a few patients, it is not the best option. In Asia, the proportion of young breast cancer patients is higher than in certain Western countries^15^,^16^.Younger patients are at a higher risk for local recurrence than older ones. Neff PT^17^ finds that the 5-year recurrence rates in patients aged below 40 and over 40 years were 24% and 6%, respectively. Similarly, Chan A found that the rate of local recurrence in patients younger than 32 years after BCS was up to 37%, while the rate of patients receiving mastectomy was only 27%^18^. Therefore, BCS may not be the best choice for young women or patients with larger tumors, for whom ENSM with immediate implant-based reconstruction is an alternative^5^,^19^.

Studies have reported complications after BCS and TSSM, including necrosis of skin, upper extremity edema, infection, seroma, and so on^9^,^10^,^11^,^12^. However, complications after ENSM are rarely reported. The incisions of ENSM are less than 2 cm except for the axillary incision that is 5 cm, compared with the incisions of BCS, which are 5–8 cm. Our study demonstrated that postoperative complication rates were low in both BCS and ENSM, without any difference between the two groups. Meanwhile, the rates of nipple necrosis and marginal necrosis of incision were 4.46% and 7%, respectively, in the ENSM group, similar to other reports^9^,^10^,^11^, indicating that ENSM was comparable to BCS in safety. Furthermore, ENSM not only yielded similar results compared with the traditional method, but also avoided obvious incisions on the breast surface, which retained the continuity of the skin. As a result, ENSM resulted in ideal breast shape more easily than BCS.

Upper limb lymphedema is a common and unpleasant complication after axillary lymph node dissection (ALND). The incidence of lymphedema ranges from 6% to 49%^20^,^21^,^22^,^23^. Vojackova^24^, Koul^25^ *et al*. found that professional massage, elastic bandages, and limb functional exercise effectively reduced the local tissue congestion, promoted lymph circulation, and partially alleviated upper limb lymphedema after ALND. However, no radical therapy is available for lymphedema. After at least 52 months of follow-up, we found that the incidence of lymphedema in the ENSM group was 9.55% (15/157), lower than in the BCS group. However, the difference was not significant. Considering that 74% of BCS (140/189) patients underwent sentinel lymph node biopsy, which was higher than in the ENSM group (33%,52/157),there were more patients avoided receiving standard axillary lymph node dissection in BCS group. To the best of our knowledge, SLND reduced the incidence of lymphedema when compared with standard axillary lymph node dissection. In this study, the ENSM group showed a lower incidence of lymphedema, which may be an endoscopic view. Comprehensive endoscopic surgery may help protect the sheath around the axillary vein and axillary vein lymph fluid circulation. Aponte-Rueda^26^,^27^,^28^ *et al*. found that the incidence of lymphedema ranged from 2.4% to 43% in traditional axillary lymph node dissection, compared with 1% to 20% in endoscopic surgery. In addition, the minor incisions of endoscopic surgery also protect the skin and subcutaneous lymph circulation. Furthermore, all the patients in the BCS group underwent postoperative radiotherapy. Radiotherapy after BCS increases the risk of lymphedema. Therefore, patients undergoing ENSM avoid postoperative radiotherapy, which may explain the lower incidence of lymphedema compared with that of BCS group (9.55% vs. 12.17%).

Radiation therapy reduces the recurrence rate in patients receiving BCS. While patients benefit from postoperative radiotherapy^29^, it can trigger serious complications such as skin damage, breast edema^30^, and radioactive pneumonia, which seriously affect patient’s health and quality of life. Patients undergoing ENSM avoid radiation therapy by removing breast tissues. Parasternal, axillary and supraclavicular radiotherapy is only provided when postoperative pathological results confirm more than three axillary lymph nodes metastases. This approach reduces the postoperative dose of radiotherapy compared with BCS and the incidence of complications after radiotherapy. In this study, radiotherapy was delivered to the breast region of all patients in the BCS group, and to the auxiliary, parasternal and supraclavicular regions in 14 cases (7.41%) of this group compared with only 18 cases (11.46%) in the ENSM group. Further, the incidence of capsular contracture in patients receiving radiation was significantly higher than in patients not treated with radiotherapy^31^,^32^. In our study, capsular contracture in most patients was mild to moderate in the ENSM group. Only 2 cases needed prosthesis removal because of intractable pain caused by severe capsular contracture, which was largely attributed to the absence of radiotherapy indication in most patients of the ENSM group.

Consistent with other reports^10^,^11^, we found no significant difference in local recurrence rate and distant metastasis between the two groups during 52 to 111 months of postoperative follow-up. We only found no patient in ENSM group with local recurrence, further establishing the safety and reliability of Endoscopic surgery. It is possibly attributed to the excision of all mammary glands, which prevents the recurrence induced by the residual gland. As reported previously, the high risk of recurrence after BCS in young breast cancer patients diminishes their quality of life due to fear of local recurrence and treatment^8^. However, patients receiving ENSM do not suffer any of these negative effects. Thus, we believe that ENSM is safe for eligible young and old patients when the delicate surgery is performed by highly experienced professionals.

Several studies have reported that autogenous tissue reconstruction was superior to prosthetic reconstruction in terms of appearance and tactile impression^33^,^34^. However, autogenous tissue reconstruction in patients with obesity, smoking and hypertension may lead to flap ischemia or necrosis, thus lowering the success rate^35^,^36^. Prosthesis reconstruction causes less trauma than autogenous tissue reconstruction, which explains the increasing popularity of prosthesis reconstruction in recent years^37^. With increased surgical expertise and strict request for inclusion criteria(patients with oversized breasts, i.e., more than 350 mL or mastoptosis, who are contraindicated for surgery), resulting in enhanced symmetry and aesthetic appeal. We found that the ENSM group also showed better cosmetic effects than the conservative surgery group. Consistent with a previous study^38^, we believe that patients of thinner stature and smaller breasts without mastoptosis are most appropriate candidates for ENSM surgery. As most East Asian women meet the surgical criteria, improved cosmetic outcomes are possible.

ENSM evolved from TSSM, ENSM took longer time than TSSM according to published literature^39^. However, incisions on the surface of breast or areola in TSSM leave a visible scar. In endoscopic surgery, the operating space is established by inflation. The amplified endoscopic view facilitates delicate surgeries, reducing intraoperative blood loss during glandular or axillary lymph node dissection. Although the operation time and economy cost is higher than the TSSM group, patients in ENSM group can benefit more from the procedure.

## Conclusion

This study shows that ENSM is a safe and effective surgery, which retains the advantages of TSSM. It further enhances the appearance of breasts without additional risks. The therapy represents an alternative treatment for early-stage breast cancer patients.

## Additional Information

**How to cite this article**: Du, J. *et al*. Endoscopic nipple sparing mastectomy with immediate implant-based reconstruction versus breast conserving surgery: a long-term study. *Sci. Rep.* **7**, 45636; doi: 10.1038/srep45636 (2017).

**Publisher's note:** Springer Nature remains neutral with regard to jurisdictional claims in published maps and institutional affiliations.

## Figures and Tables

**Table 1:** Operation details and clinical pathology feature of two groups.

Parameter	BCS	ENSM	p value
Cases	189	157	
Age			0.204
≤40 years	68	67	
>40 years	121	90	
TNM stage			0.052
I	70	36	
IIA	81	65	
IIB	24	32	
IIIA	15	24	
ER			0.911
Postive	135	113	
Negative	54	44	
HER-2			0.851
Postive	62	53	
Negative	127	104	
pathological types			0.183
Invasive Ductal Carcinoma	165	129	
Other types	24	28	
Operation time (mean, min)	165	197	0.001
Blood loss (mean, ml)	83	95	0.134
drainage (mean, ml)	230	250	0.384
sentinel lymph node biopsy	140	52	0.000
Metastasis lymph node number			0.195
≤3	175	139	
>3	14	18	

**Table 2:** Comparison of postoperative complication between two groups.

Parameter	BCS	ENSM	p value
Infection	8 (4.23%)	5 (3.18%)	0.610
marginal necrosis of incison	16 (8.46%)	11 (7.01%)	0.534
Upper limb lymphedema	23 (12.16%)	15 (9.55%)	0.439

**Table 3:** Comparison of cosmetic effect between two groups.

Parameter	BCS	ENSM	p value
Excellent	34 (17.99%)	29 (18.47%)	0.161
Good	76 (40.21%)	80 (50.96%)	
Mediocre	57 (30.16%)	35 (22.29%)	
Poor	22 (11.64%)	13 (8.28%)	

## References

[b1] Chen W., Zheng R., Zhang S., Zhang S. & He J. Annual report on status of cancer in China, 2011. J. Chin J Cancer Res. 27(1), 2–12 (2015). 25717220 10.3978/j.issn.1000-9604.2015.01.06 PMC4329176

[b2] DeSantis C. E. . Cancer treatment and survivorship statistics, 2014. J CA Cancer J Clin. 64(4), 252–71 (2014). 24890451 10.3322/caac.21235

[b3] Howlader N. . SEER Cancer Statistics Review, 1975–2010. Bethesda, MD: National Cancer Institute http://seer.cancer.gov/csr/1975_2010 (2013).

[b4] Veronesi U. . Twenty-year follow up of a randomized study comparing breast-conserving surgery with radical mastectomy for early breast cancer. J.N Engl J Med. 347, 1227–32 (2002). 12393819 10.1056/NEJMoa020989

[b5] McGuire K. P. . Are mastectomies on the rise? A 13-year trend analysis of the selection of mastectomy versus breast conservation therapy in 5865 patients. J. Ann Surg Oncol. 16(10), 2682–90 (2009). 10.1245/s10434-009-0635-x 19653046

[b6] Li J. . A nation-wide multicenter 10-year (1999–2008) retrospective clinical epidemiological study of female breast cancer in China. J.BMC Cancer. 11, 364 (2011). 10.1186/1471-2407-11-364 PMC3178543 21859480

[b7] Yuan X. M. . Current status of diagnosis and treatment of primary breast cancer in Beijing. J. Chin J Cancer Res 2011 23, 38–42 (2008). 10.1007/s11670-011-0038-y PMC3587532 23467615

[b8] Zhang B. N. & Zhang H. M. Some questions should be noticed in breast conserve surgery in our country. J. chinese medicine journal. 88(02), 73–76 (2008).

[b9] Piper M., Peled A. W., Foster R. D., Moore D. H. & Esserman L. J. Total Skin-Sparing Mastectomy: A Systematic Review of Oncologic Outcomes and Postoperative Complications. J. Ann Plast Surg. 701(4),435–7 (2013). 10.1097/SAP.0b013e31827e5333 23486127

[b10] Stanec Z. . Skin and nipple-areola complex sparing mastectomy in breast cancer patients: 15-year experience. J. Ann Plast Surg. 73(5), 485–91 (2013). 10.1097/SAP.0b013e31827a30e6 24378808

[b11] Munhoz A. M. . Clinical outcomes following nipple-areola-sparing mastectomy with immediate implant- based breast reconstruction: a 12-year experience with an analysis of patient and breast-related factors for complications. J.Breast Cancer Res Treat. 140, 545–55 (2013). 10.1007/s10549-013-2634-7 23897416

[b12] Mallon P. . The role of nipple sparing mastectomy in breast cancer: a comprehensive review of the liter- ature. J Plast Reconstr Surg. 131(5), 969–84 (2013). 10.1097/PRS.0b013e3182865a3c 23629079

[b13] Mansel R. E. . Randomized multicenter trial of sentinel node biopsy versus standard axillary treatment in operable breast cancer: the ALMANAC Trial. J. Natl Cancer Inst. 98, 599–609 (2006). 16670385 10.1093/jnci/djj158

[b14] Gill G. & SNAC Trial Group of the Royal Australasian College of Surgeons (RACS) and NHMRC Clinical Trials Centre. Sentinel- lymph-node-based management or routine axillary clearance? One-year outcomes of sentinel node biopsy versus axillary clearance (SNAC): a randomized controlled surgical trial. [J] Ann Surg Oncol. 16, 266–75 (2009). 10.1245/s10434-008-0229-z 19050973

[b15] Agarwal G., Pradeep P. V., Aggarwal V., Yip C. H. & Cheung P. S. Spectrum of breast cancer in Asian women. J. World J Surg. 31, 1031–40 (2007). 17387549 10.1007/s00268-005-0585-9

[b16] Bantema-Joppe E. J. . Early-stage young breast cancer patients: impact of local treatment on survival. J. Int J Radiat Oncol Biol Phys. 81(4), e553–9 (2011). 21601378 10.1016/j.ijrobp.2011.02.060

[b17] Neff P. T. . Long-term results of breast conservation therapy for breast cancer. J. Ann Surg. 223, 709–17 (1996). 10.1097/00000658-199606000-00009 PMC1235217 8645044

[b18] Chan A., Pintilie M., Vallis K., Girourd C. & Goss P. Breast cancer in women B35 years: review of 1002 cases from a single institution. J. Ann Oncol. 11, 1255–62 (2000). 10.1023/a:1008391401404 11106113

[b19] Lee C. N. . Are patients making high-quality decisions about breast reconstruction after mastectomy? [outcomes articles]. J. Plast Reconstr Surg. 127, 18–26 (2011). 10.1097/PRS.0b013e3181f958de PMC4100583 21200195

[b20] Pavlista D. & Eliska O. Analysis of direct oil contrast lymphography of upper limb lymphatics traversing the axilla –a lesson from the past -contribution to the concept of axillary reverse mapping. J. Eur J Surg Oncol. 38(5), 390–394 (2012). 22336143 10.1016/j.ejso.2012.01.010

[b21] Stanton A. W. . Lymphatic drainage in the muscle and subcutis of the arm after breast cancer treatment. J. Breast Cancer Res Treat. 117(3), 549–557 (2009). 10.1007/s10549-008-0259-z 19052859

[b22] Fisher B. . Ten-year results of a randomized clinical trial comparing radical mastectomy and total mastectomy with or without radiation. J. N Engl J Med. 312(11), 674–681 (1985). 3883168 10.1056/NEJM198503143121102

[b23] Petrek J. A., Senie R. T., Peters M. & Rosen P. P. Lymphedema in a cohort of breast carcinoma survivors 20 years after diagnosis. J. Cancer. 92(6), 1368–1377 (2001). 10.1002/1097-0142(20010915)92:6<1368::aid-cncr1459>3.0.co;2-9 11745212

[b24] Vojácková N., Sebková M., Schmiedbergerová R. & Hercogová J. Cohort of lymphedema patients followed at the Lymphology Centre of the Dermatovenerologcal Clinic of the 2nd Faculty of Medicine Univemity Hospital Na Bulovce Prague in years 2000 to 2005.A retrospective study. J. Cas Lek Cesk. 146(1), 57–61 (2007). 17310586

[b25] Koul R. . Efficacy of complete decongestive therapy and manual lymphatic drainage on treatment-related lymphedema in breast cancer. J. Int J Radiat Oncol Biol Phys. 67(3), 841–846 (2007). 17175115 10.1016/j.ijrobp.2006.09.024

[b26] Aponte-Rueda M. E., Saade Cárdenas R. A. & Saade Aure M. J. Endoscopic axillary dissection: a systematic review of the literature. J.Breast. 18(3), 150–8 (2009). 10.1016/j.breast.2009.05.001 19493679

[b27] Sehrenk P., Rieger R., Shamiyeh A. & Wayand W. Morbidity following sentinel lymph node biopsy versus axillary lymph node dissection for patients with breast carcinoma. J. Cancer. 88, 608–614 (2000). 10.1002/(sici)1097-0142(20000201)88:3<608::aid-cncr17>3.0.co;2-k 10649254

[b28] Hinrichs C. S. . Lymphedema secondary to postmastectomy radiation: incidence and risk factors. J.Ann Surg Oncol. 11(6), 573–80 (2004). 10.1245/ASO.2004.04.017 15172932

[b29] Darby S. . Effect of radiotherapy after breast-conserving surgery on 10-year recurrence and 15-year breast cancer death: meta-analysis of individual patient data for 10,801 women in 17 randomised trials. J. Lancet. 378, 1707–1716 (2011). 10.1016/S0140-6736(11)61629-2 PMC3254252 22019144

[b30] Van Limbergen E. & Weltens C. New trends in radiotherapy for breast cancer. J. Curr Opin Oncol. 18, 555–562 (2006). 10.1097/01.cco.0000245327.42281.9f 16988575

[b31] Benediktsson K. & Perbeck L. Capsular contracture around salinefilled and textured subcutaneously-placed implants in irradiated and non-irradiated breast cancer patients: five years of monitoring of a prospective trial. J. J Plast Reconstr Aesthet Surg. 59(1), 27–34 (2006). 16482787 10.1016/j.bjps.2005.08.005

[b32] Barry M. & Kell M. R. Radiotherapy and breast reconstruction: a meta-analysis. J. Breast Cancer Res Treat. 127, 15–22 (2011). 10.1007/s10549-011-1401-x 21336948

[b33] Liu C. . Quality of life and patient satisfaction after microsurgical abdominal flap versus staged expander/implant breast reconstruction: a critical study of unilateral immediate breast reconstruction using patient-reported outcomes instrument BREAST-Q. J. Breast Cancer Res Treat. 146(1), 117–26 (2014). 10.1007/s10549-014-2981-z 24831775

[b34] Tønseth K. A., Hokland B. M., Tindholdt T. T., Abyholm F. E. & Stavem K. Quality of life, patient satisfaction and cosmetic outcome after breast reconstruction using DIEP flap orexpandable breast implant. J. J Plast Reconstr Aesthet Surg. 61(10), 1188–94 (2008). 17604241 10.1016/j.bjps.2007.05.006

[b35] Gill P. S. . A 10-year retrospective review of 758 DIEP flaps for breast reconstruction. J. Plast Reconstr Surg. 113, 1153 (2004). 10.1097/01.prs.0000110328.47206.50 15083015

[b36] Mehrara B. J. . Complications after microvascular breast reconstruction: Experience with 1195 flaps. J. Plast Reconstr Surg. 118, 1100 (2006) 10.1097/01.prs.0000236898.87398.d6 17016173

[b37] Albornoz C. R. . A paradigm shift in U.S. Breast reconstruction: increasing implant rates. J. Plast Reconstr Surg.Jan. 131(1), 15–23 (2013). 10.1097/PRS.0b013e3182729cde 23271515

[b38] Disa J. J. Breast Reconstruction Prosthetic Techniques in Grabb & Smith’s Plastic Surgery(ed. Disa J. J.) 625–633 (Lippincott Williams & Wilkins, 2007).

[b39] Satoki Kinoshita. . Clinical comparison of four types of skin incisions for skin-sparing mastectomy and immediate breast reconstruction. J. Surg Today. 44, 1470–1475 (2014). 10.1007/s00595-013-0722-2 PMC4097197 24043394

